# A Laser-Based Multipass Absorption Sensor for Sub-ppm Detection of Methane, Acetylene and Ammonia

**DOI:** 10.3390/s22020556

**Published:** 2022-01-12

**Authors:** Wei Duan, Fuwu Yan, Yu Wang, Hui Zhang, Liuhao Ma, Daxin Wen, Wei Wang, Gang Sheng, Qiang Wang

**Affiliations:** 1 Hubei Key Laboratory of Advanced Technology for Automotive Components, School of Automotive Engineering, Wuhan University of Technology, Wuhan 430070, China; wei.duan@whut.edu.cn (W.D.); yanfuwu@vip.sina.com (F.Y.); yu.wang@whut.edu.cn (Y.W.); wendaxin@whut.edu.cn (D.W.); wwang1561988@whut.edu.cn (W.W.); shenggang@whut.edu.cn (G.S.); 2 Key Laboratory of Power Machinery and Engineering of Ministry of Education, Shanghai Jiao Tong University, Shanghai 200240, China; 3 State Key Laboratory of Applied Optics, Changchun Institute of Optics, Fine Mechanics and Physics, Chinese Academy of Sciences, Changchun 130033, China; zhanghui195@mails.ucas.ac.cn

**Keywords:** multispecies sensor, sensitive detection, wavelength modulation spectroscopy, optical sensor

## Abstract

A compact, sensitive laser-based absorption sensor for multispecies monitoring of methane (CH_4_), acetylene (C_2_H_2_) and ammonia (NH_3_) was developed using a compact multipass gas cell. The gas cell is 8.8 cm long and has an effective optical path length of 3.0 m with a sampling volume of 75 mL. The sensor is composed of three fiber-coupled distributed feedback lasers operating near 1512 nm, 1532 nm and 1654 nm, an InGaAs photodetector and a custom-designed software for data acquisition, signal processing and display. The lasers were scanned over the target absorption features at 1 Hz. First-harmonic-normalized wavelength modulation spectroscopy (*f* = 3 kHz) with the second harmonic detection (WMS-2*f*/1*f*) is employed to eliminate the unwanted power fluctuations of the transmitted laser caused by aerosol/particles scattering, absorption and beam-steering. The multispecies sensor has excellent linear responses (R^2^ > 0.997) within the gas concentration range of 1–1000 ppm and shows a detection limit of 0.32 ppm for CH_4_, 0.16 ppm for C_2_H_2_ and 0.23 ppm for NH_3_ at 1 s response time. The Allan–Werle deviation analysis verifies the long-term stability of the sensor, indicating a minimal detection limit of 20–34 ppb were achieved after 60–148 s integration time. Flow test of the portable multispecies sensor is also demonstrated in this work.

## 1. Introduction

Accurate knowledge of trace gas concentration is crucial for inflammable, explosive and poisonous gas leakage warning [1], medical breath diagnostics [2], environmental monitoring [3,4,5] and active control of combustion and propulsion systems [6,7]. The worldwide target for “Peak carbon dioxide emission” and “Carbon neutrality” further emphasizes the significance of trace gas sensing, particularly for greenhouse gases and atmospheric pollutants [8,9]. Methane (CH_4_), a representative greenhouse gas, mainly comes from anthropogenic and natural sources such as production and transport of fossil fuels and agricultural practices [10]. Another light hydrocarbon C_2_H_2_ is widely known as a molecular growth agent towards large polycyclic aromatic hydrocarbons (PAH) precursor for soot formation and thus plays a very important role in soot formation [11]. Note that C_2_H_2_ is a typical combustion intermediate during incomplete combustion of hydrocarbon fuels [12].

Recently, NH_3_ has been recognized as a potential carbon-neutral fuel that can help to accelerate the reduction of greenhouse gas emissions [13]. However, NH_3_ is a prevalent atmospheric pollutant and its leakage would lead to the formation of ammonium salt, which will further contribute to the formation of particulate matter. Additionally, for stationary emission sources such as thermal power plant, NH_3_ is used as a reductant to convert the NO_x_ pollutant to N_2_. In practical applications, excessive spray of NH_3_ could be used which may cause the ammonia to escape. Therefore, precise and sensitive measurement of CH_4_, C_2_H_2_ and NH_3_ is crucial to not only the environmental monitoring but the evaluation of the combustion process.

Over the past decades, various techniques have been developed for quantitative measurements of gas concentrations, including electrochemical method [14], Fourier transform spectroscopy [15], non-dispersive infrared method [16], laser-based absorption and dispersion spectroscopy [17,18,19,20], photoacoustic and photothermal spectroscopy [21,22]. Many of these techniques have been transferred to commercially-available sensors and have been continuously improved to satisfy the increasing demand for higher detectivity, sensitivity and robustness. Among these techniques, laser absorption spectroscopy (LAS) represents one of the most widely-used and reliable methods due to its fast response, species-selectivity, high sensitivity, detectivity, robustness and relatively simple optical configuration [23].

For LAS-based sensors, direct absorption spectroscopy (DAS) and wavelength modulation spectroscopy (WMS) are two typical measurement schemes. DAS is a straightforward technique for gas detection. By baseline fitting to the transmitted absorption profile, the gas concentration can be quantified. However, the unexpected laser power fluctuations induced by laser systems and particles scattering/absorption from the target environment will definitely introduce unwanted interferences and extra uncertainties. Additionally, the detection limit of DAS is limited due to low-frequency noises. Note that WMS method overcomes the baseline fitting issue and improves the detectivity by shifting the detection bandwidth to higher frequencies (twice the modulation frequency) [24]. The WMS method with second harmonic signal (2*f*) detection (WMS-2*f*) has been widely used for trace gas sensing. However, the 2*f* signal magnitude will be influenced by the laser power fluctuations in addition to being sensitive to the photodetector shifts. By normalizing the second harmonic signal by the first harmonic signal which is simultaneously recorded (i.e., WMS-2*f*/1*f*), the influence of optical power fluctuations can be largely eliminated [25,26,27,28]. Hence, the WMS-2*f*/1*f* method is favorable for trace gas detection.

Recent technical advancements of multipass gas cell and the mature telecommunication laser technologies provide a promising solution for developing trace gas sensors using a combination of distributed feedback (DFB) lasers and compact multipass gas cell [29,30,31]. Many efforts have been devoted to developing trace gas sensors using such kind of configurations. More specifically, Liu et al. developed a highly sensitive WMS-based CH_4_ sensor using a compact dense-pattern multipass cell for atmospheric CH_4_ measurement [32]. A measurement precision of <79 ppb per second was achieved and a minimum detection limit of 15 ppb can be reached with an integrated time of 70 s. Shao et al. presented a compact sensor using a single DFB laser near 2.3 μm for simultaneous detection of CO and CH_4_ [33]. The sensor has a detectivity of ppb level with over two minutes averaging and has the advantage of high robustness which is achieved through utilizing the WMS-2*f*/1*f* method. Sun et al. developed a highly sensitive acetylene sensor using optimized WMS technique and compact multipass cell [34]. Jin et al. reported a robust fiber-coupled sensor for remote dual-species measurement of CH_4_ and C_2_H_2_ using a combination of frequency-division multiplexing method and WMS-2*f*/1*f* [35]. Sub-ppm sensitivity with 1s time resolution was achieved for both gas species. Guo et al. developed a portable sensor for in situ measurement of NH_3_ and applied the sensor for monitoring the flue gas of coke oven after Selective Catalytic Reduction (SCR) System [36]. Li et al. developed a portable and robust LAS-based sensor for dual species (i.e., NH_3_ and H_2_O) measurements in the denitrification processes of thermal power plants [37]. Bai et al. proposed the development of a NH_3_ detection system with wide dynamics range based on numerical simulation [38]. Raza et al. performed simultaneous measurement of NH_3_ and CO at elevated temperature. At the temperature of 700 K and absorption length of 20 cm, the noise equivalent absorption (NEA) coefficient of 3.5 × 10^−6^ cm^−1^ for CO and 4.9 × 10^−7^ cm^−1^ for NH_3_ was achieved [39]. Note that, the commercially-available mid-infrared (MIR), room temperature laser sources make it accessible to target the strong absorption features in the fundamental band. Although more sensitive sensors can be developed using the mid-infrared laser sources, the MIR lasers are too costly to be frequently and extensively used in practical applications. By implementing the low-cost DFB lasers and portable multipass gas cell, laser sensor with desired sensitivity and detectivity can be also developed with promising application potentials.

In this work, we report a sensitive sensor for simultaneous trace-gas detection of CH_4_, C_2_H_2_ and NH_3_ using multiple NIR DFB lasers and a compact multipass gas cell. Precise line selection was performed to select the appropriate line for detection. WMS-2*f*/1*f* strategy was adopted to achieve low-detection limit and high precision. The sensor shows a sub-ppm minimum detection limit (MDL) at the response time of 1.0 s for CH_4_, C_2_H_2_ and NH_3_, respectively. The lower detection limit can be further improved to 30–50 ppb by averaging up to 100 s. Flow test of the developed sensor under different gas concentrations was performed in this work, proving its high practicability to real-life applications.

## 2. Spectroscopic Fundamentals

The principle of laser absorption spectroscopy (LAS) has been well documented in previous literature [40]. Here, only a brief description is reproduced to clarify the nomenclature and abbreviations used in the current work. When the collimated, monochromatic laser radiation interacts with the target gas molecules in the vicinity of molecular resonance within a homogeneous environment of length *L* (cm), the fractional attenuation of incident radiation intensity is governed by the Beer–Lambert relation [40]:

(1) $$\alpha_{v}=-\ln{\left(\frac{I_{t}}{I_{0}}\right)}_{v}=k_{v}L=S_{i}(T)\phi_{v}PX_{abs}L$$

where *α_v_* and *k_v_* (cm^−1^) are the frequency-dependent absorbance and absorption coefficient at the optical frequency *v* (cm^−1^), respectively; *I_0_* and It indicate the intensities of the incident and the transmitted laser, respectively; *P* (atm) denotes the total gas pressure, *S_i_*(*T*) (cm^−2^ atm^−1^) is the temperature dependent line-strength of the specific transition *i*, *ϕ_v_* (cm) represents the normalized line-shape function, *T* and *X_abs_* are the temperature and species concentration of the absorption gas of interest.

For trace gas sensing, wavelength modulation spectroscopy with second harmonic detection (WMS-2*f*) is a well-established LAS technique and has been widely used in lab-scale and practical environments due to its high signal-to-noise ratio (SNR). This method eliminates the low-frequency noises by shifting the detection bandwidth to higher frequencies (>kHz), which is above the frequency range of many common noise sources [41]. Note that the improved first-harmonic-normalized wavelength modulation spectroscopy with the second harmonic detection (WMS-2*f*/1*f*) is another alternative and promising method that eliminates the low-frequency noises. By normalization of the 2*f* signal by the simultaneous 1*f* detection, the 2*f*/1*f* signal can be made immune to the spurious transmitted laser power fluctuations caused by aerosol/particles scattering, absorption and beam-steering.

The WMS-2*f* includes modulating the laser injection current near the absorption feature of the target species and isolating the 2*f* signal by a lock-in amplifier. The peak height of the 2*f* signal is used to infer gas properties. The modulation involves a slow linear scan and a rapid sinusoidal dither. The frequency v(t) and intensity I(t) of the incident laser can be expressed by:

(2) $$v(t)=\bar{v}(t)+a\cos(\omega t)$$

(3) $$I_{0}(t)={\bar{I}}_{0}(t)[1+i_{0}(t)\cos(\omega t+\psi_{1})+i_{2}(t)\cos(2\omega t+\psi_{2})]$$

where $\bar{v}(t)$ (cm^−1^) is the laser center frequency of the scan signal, a (cm^−1^) is the modulation depth, ω is the modulation frequency, $\bar{I_{0}}(t)$ is the averaged laser intensity, $i_{0}(t)$ is the intensity modulation amplitude normalized by $\bar{I_{0}}(t)$, $i_{1}$ and $i_{2}$ represent the amplitudes of linear and non-linear intensity modulation, $\psi_{1}$ and $\psi_{2}$ denote the linear and non-linear phase shift between frequency modulation and intensity modulation.

When the modulated laser beam passes through the target species, the transmitted laser intensity *I_t_*(*t*) is given by:

(4) $$I_{t}(t)=I_{0}(t)\cdot \tau (\bar{v}+a\cos\omega t)$$

where $\tau (\bar{v}+a\cos\omega t)$ is the transmission coefficient. The transmission coefficient can be expanded in a Fourier cosine series:

(5) $$\tau (\bar{v}+a\cos\omega t)=\sum_{k=0}^{\infty }H_{k}(\bar{v},a)\cdot \cos(k\omega t)$$

The magnitude of the 2*f* and 1*f* signal become:

(6) $$S_{2f}=\frac{G{\bar{I}}_{0}}{2}{\left\{{\left[H_{2}+\frac{i_{0}}{2}(H_{1}+H_{3})\cos\psi_{1}\right]}^{2}+{\left[\frac{i_{0}}{2}(H_{1}-H_{3})\sin\psi_{1}\right]}^{2}\right\}}^{1/2}$$

(7) $$R_{1f}=\frac{G{\bar{I}}_{0}}{2}{\left\{{\left[H_{1}+i_{0}(H_{0}+\frac{H_{2}}{2})\cos\psi_{1}\right]}^{2}+{\left[i_{0}(H_{0}-\frac{H_{2}}{2})\sin\psi_{1}\right]}^{2}\right\}}^{1/2}$$

With detailed knowledge of the specific laser tuning characteristics and spectral properties of the selected absorption transitions, the 2*f*/1*f* signal can be obtained by directly calculating the Fourier coefficient of the transmission coefficient and the phase shift between frequency modulation. Therefore, the simulated 2*f*/1*f* signal reflects the experimental details. For the trace gas sensing with known temperature and pressure, gas concentration can be obtained.

## 3. Sensor Configurations

**Figure 1:**
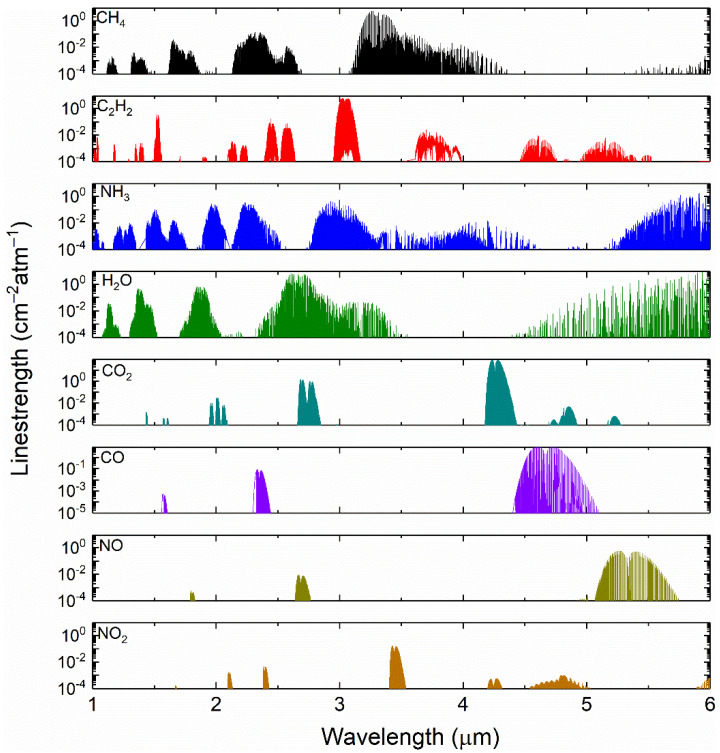
Absorption features of typical exhaust mixtures within near- and mid-infrared region (1–6 μm) at 296 K based on HITRAN 2020 database [42].

Optimal selection of absorption lines is essential to sensor design. As illustrated in Figure 1, the target species have wide absorption spectrum within the overtone, combination and fundamental bands from near-infrared to mid-infrared region. It is evident that there exist potential interferences from other species such as H_2_O, CO, CO_2_ and NO_x_. Therefore, the first priority is to find absorption lines that have good spectral isolation from the interfering gas species. In addition, sufficient line-strength should be ensured. For a minimum detectable absorbance of 0.01%, an effective absorption length of 3.0 m and required SNR of over 10 at ambient temperature and atmospheric pressure (*T* = 296 K, *P* = 1 atm), the line-strength should ensure the peak absorbance of 0.001. Considering the criteria mentioned above, we finally select absorption line near 6046.95 cm^−1^, 6534.36 cm^−1^ and 6612.70 cm^−1^ for detection of CH_4_, C_2_H_2_ and NH_3_, respectively.

**Figure 2:**
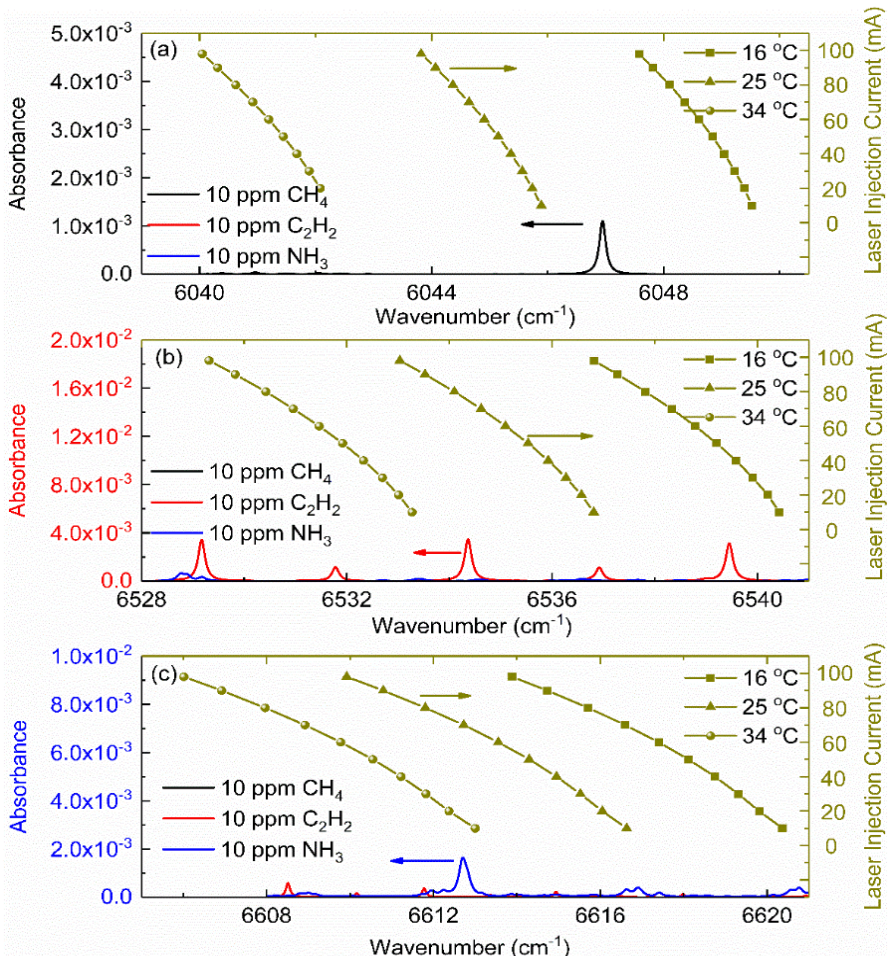
Characterization of the three DFB lasers’ tuning range on top of the target absorption line of CH_4_ (**a**), C_2_H_2_ (**b**) and NH_3_ (**c**). The condition spectral simulation is set to be *L* = 3 m, *P* = 1 atm, *T* = 298.15 K, *X*_abs_ = 10 ppm.

Three continuous-wave (CW), narrow linewidth (<3 MHz), fiber-coupled DFB lasers (NTT Electronics) mounted in butterfly packages were used as the light sources to target the selected absorption lines of CH_4_, C_2_H_2_ and NH_3_. The laser tuning performance of the emission wavelength were characterized using a spectral analyzer (Yokogawa, AQ6370). Figure 2 depicts the measured wavenumber as a function of the injection current at several representative operation temperatures (16–34 °C) and the simulated absorption spectrum within the tuning range. The three DFB lasers can be tuned over the wavenumber range 6040–6049 cm^−1^, 6529–6540 cm^−1^ and 6606–6620 cm^−1^, respectively. As illustrated in Figure 2, the absorption features of the selected lines can be precisely covered by adjusting the operation temperature and scanning the injection current simultaneously. The spectral simulations were performed based on the HITRAN 2020 database [42] at ambient temperature, atmospheric pressure, concentration of 10 ppm and optical path length of 3 m. As can be noted, the peak absorbances for the selected absorption lines are larger than 0.001 for sensitive detection using the wavelength modulation spectroscopy strategy. Additionally, it is evident to see that there exists no absorption interference from the other two species. The selected absorption line satisfies the measurement requirements.

**Figure 3:**
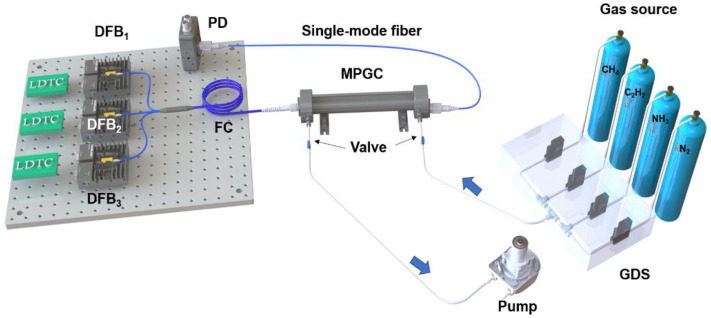
Schematic of the laser-based multipass absorption sensor. DFB, distributed feedback laser; LDTC, laser driver and temperature controller; PD, photodetector; FC, fiber coupler; MPGC, multipass gas cell; GDS, gas dilution system.

Figure 3 depicts the optical configurations of the laser-based multispecies sensor. The operation temperature and injection current of the three near-infrared DFB lasers are precisely controlled by commercially-available low noise controllers (Stanford Research Systems, LDC501). A LabVIEW-based program with a data acquisition card (National Instruments, USB 6356) was used to generate the desired triangle-scan signals and sinusoidal-modulation signals, to record the signals from photodetectors and to perform the signal trigger synchronization. The three incident laser beams were coupled as one and directed to a compact multipass gas cell (MPGC) with an enclosed volume of 75 mL. The MPGC includes two high-reflectivity quartz mirrors that are placed 8.8 cm apart from each other to have an effective absorption path length of 3.0 m. The combined laser beam then interacts with the target gas molecules. The transmitted laser beams were then delivered to the InGaAs photodetector (Thorlabs, PDA20CS2). A digital lock-in filter coupled with a low-pass filter with cutoff frequency at 10 fs (frequency of scan, i.e., 10 kHz) was applied to the raw detector signals to extract the harmonics of the WMS line shapes for each laser.

For the experiments, reference standard gas is required. The concentrations of CH_4_, C_2_H_2_ and NH_3_ cylinders are calibrated to be 1000 ppm and then diluted to gas samples with different concentrations using a highly accurate gas dilution system (LNI Swissgas, SONIMIX 7100). For the static experiments, the MPGC is pumped to vacuum by a mechanical pump and filled with the gas samples to ambient pressure at least three times. Note that all the measurements were performed at the ambient pressure of 1 atm.

## 4. Results and Discussion

**Figure 4:**
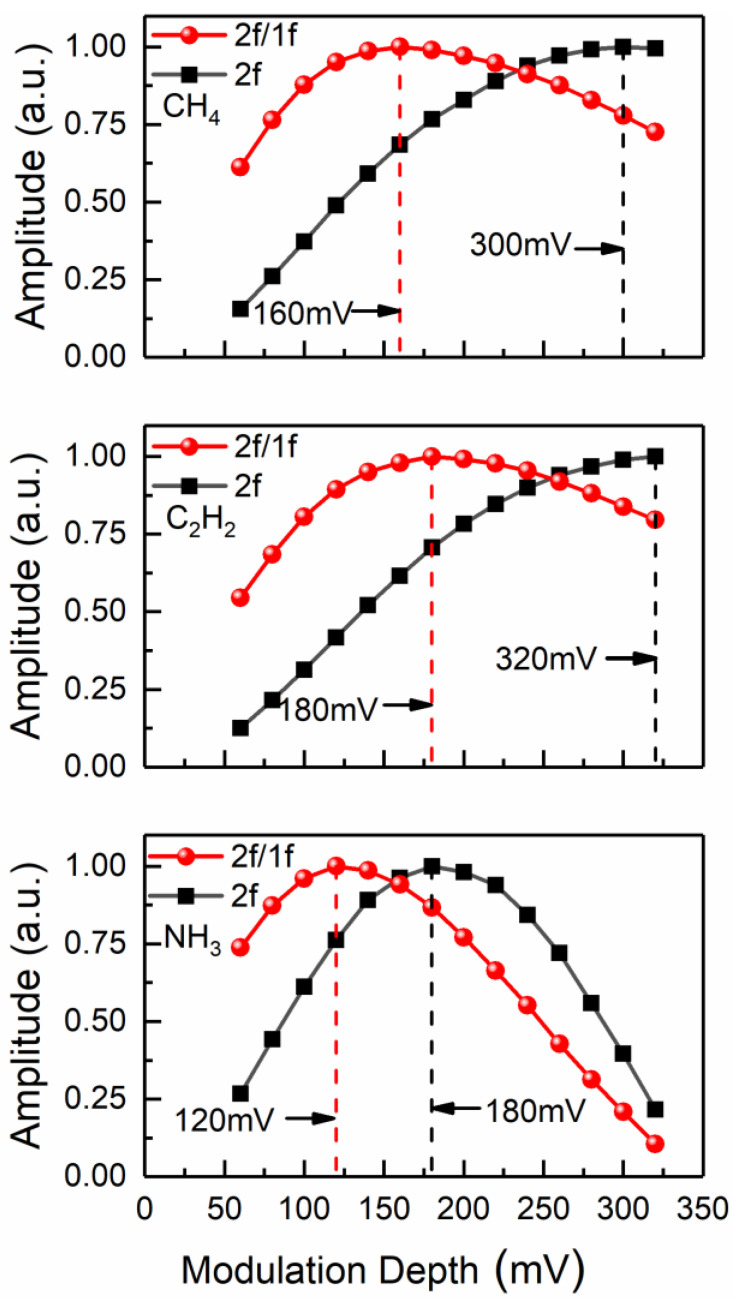
Normalized signal amplitude as a function of modulation depth.

First-harmonic-normalized wavelength modulation spectroscopy with the second harmonic detection (WMS-2*f*/1*f*) was used for sensitive detection of multiple species. Each DFB laser was scanned to cover the selected absorption feature by a 1 Hz ramp waveform. A sinusoidal dither at 3 kHz was superimposed to modulate the laser. For a fixed gas pressure, modulation frequency and a certain absorption feature, the magnitude of harmonic signals will be influenced by modulation depth. The multipass cell filled with a gas mixture sample of 100 ppm CH_4_, 100 ppm C_2_H_2_ and 100 ppm NH_3_ was used for searching the optimal modulation parameters by varying the peak-to-peak voltage of the sinusoidal modulation signal. Figure 4 shows the amplitude of 2*f* and 2*f*/1*f* signal as a function of modulation depth (in mV) for three different gases. For any specific gas, the amplitudes of both signals experience an increase and subsequent decrease when the modulation depth increase from 60 mV to 320 mV. For sensitive detection, the modulation depths were finally selected to be 160 mV, 180 mV and 120 mV for CH_4_, C_2_H_2_ and NH_3_, respectively.

**Figure 5:**
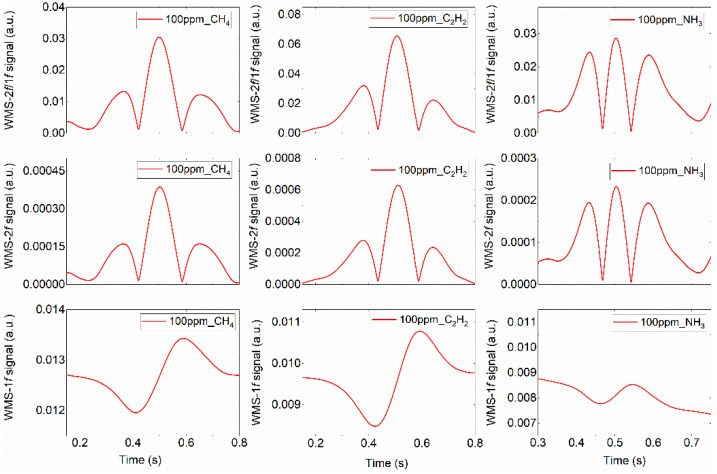
Representative WMS-2*f*/1*f*, WMS-2*f* and WMS-1*f* signals for 100 ppm CH_4_, C_2_H_2_ and NH_3_ concentrations.

With the above-mentioned optimal modulation parameters, the sensor performance was comprehensively investigated by measuring the varied gas concentrations inside the multipass cell. Figure 5 depicts the representative WMS-2*f*/1*f*, WMS-2*f* and WMS-1*f* signal profiles for gas concentrations of 100 ppm. High-fidelity signal can be clearly observed. Note that for the harmonic signals of NH_3_, there exist some raised peaks near the main peak, which is due to certain adjacent absorption lines.

**Figure 6:**
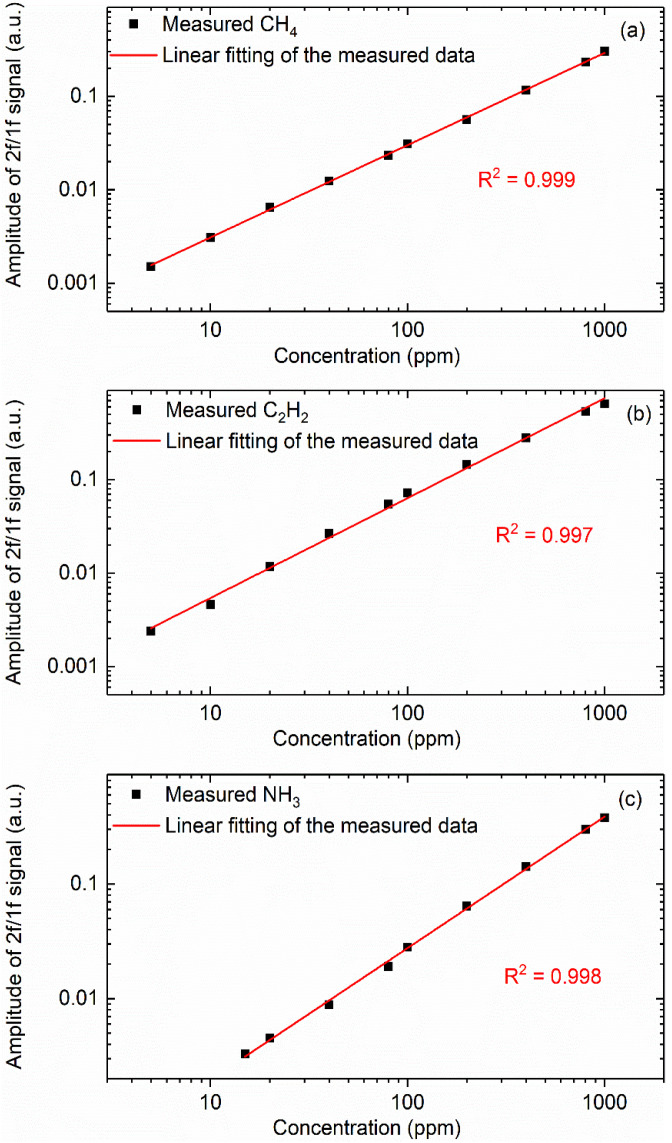
Measured amplitude of 2*f/1f* signal as a function of the gas concentration: (**a**) CH_4_, (**b**) C_2_H_2_, (**c**) NH_3_.

The measured amplitude of WMS-2*f*/1*f* signals at different CH_4_, C_2_H_2_ and NH_3_ concentrations were plotted in Figure 6. The solid lines represent the linear fitting to the experimentally-measured peak amplitude of the harmonic signals. All the R-square values for the linear fitting are estimated to be over 0.997, indicating an excellent linear response of the developed multispecies gas sensor. The linear fitting line can be used to estimate the detection limits of the current sensor by Allan–Werle deviation analysis, which will be elaborated.

**Figure 7:**
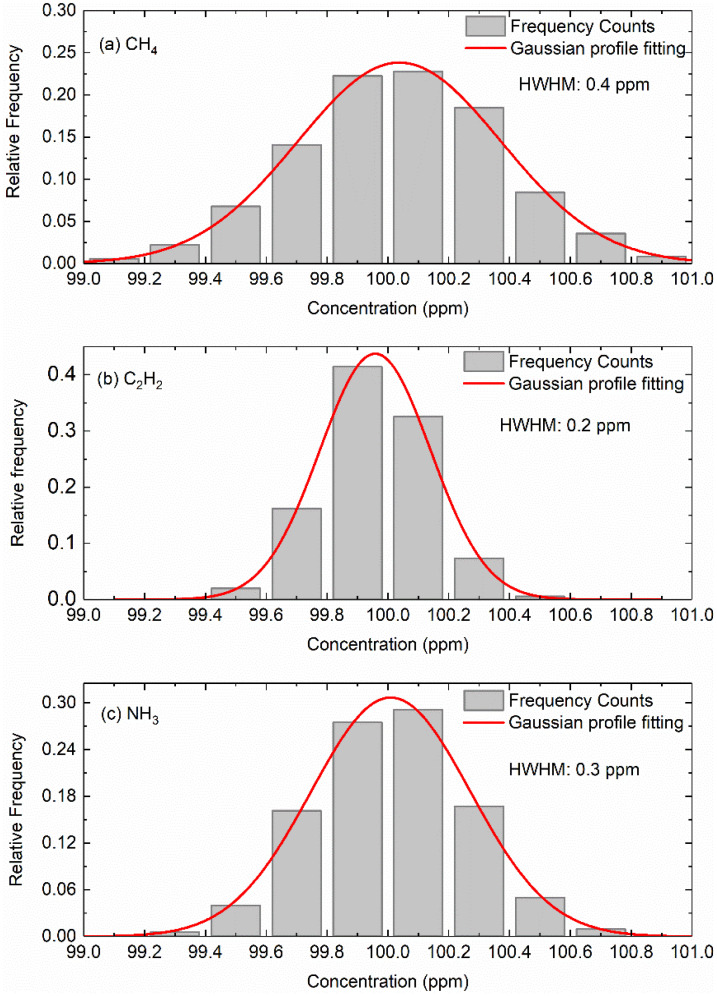
Frequency distribution of the measured data along with the Gaussian function fitting based on the time-resolved measurements of 100 ppm CH_4_ (**a**), 100 ppm C_2_H_2_ (**b**) and 100 ppm NH_3_ (**c**) in the multipass gas cell.

To further evaluate the precision of current measurements, continuous measurements of 100 ppm CH_4_, C_2_H_2_ and NH_3_ were performed at static environment for 30 min. Figure 7 presents the frequency distribution (shown in histogram plot) of the measured data. It is clearly seen that the frequency distribution can be fitting by a Gaussian profile. The half width at half maximum (HWHM) of 0.4 ppm, 0.2 ppm and 0.3 ppm for CH_4_, C_2_H_2_ and NH_3_, corresponding to a relative instrument precision level of ~0.5%.

**Figure 8:**
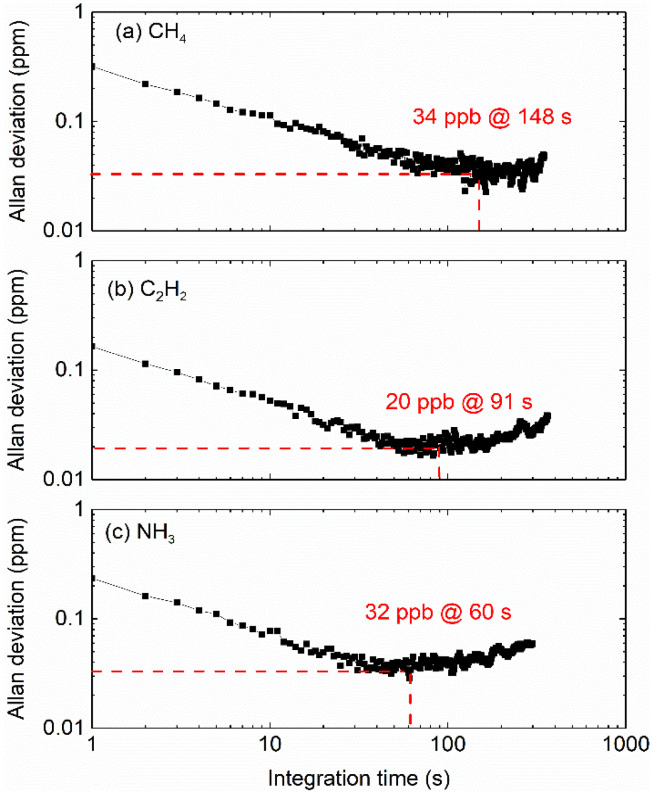
The Allan–Werle deviation curve of the current sensor for CH_4_ (**a**), C_2_H_2_ (**b**) and NH_3_ (**c**).

In additional to precision evaluation, Allan–Werle deviation analysis were also performed to estimate the long-term stability of the sensor. The measurement detection limit was analyzed by evaluating the time-resolved variations of the 2*f*/1*f* signal for gas concentrations of 100 ppm. It should be noted that the 1σ-noise voltage can be converted to gas concentration by exploiting the calibration curve illustrated in Figure 6 and time-resolved gas concentrations can be also directly used to get the Allan–Werle deviation curve. At a time resolution of 1 s, the MDL of CH_4_, C_2_H_2_ and NH_3_ were determined to be 0.32 ppm, 0.16 ppm and 0.23 ppm for an effective absorption path length of 3.0 m. It should be noted that the MDL can be further improved with the increase of integration time. As illustrated in Figure 8, at the measurement time of 60–100 s, the sensor achieved an MDL of 20–34 ppb.

**Figure 9:**
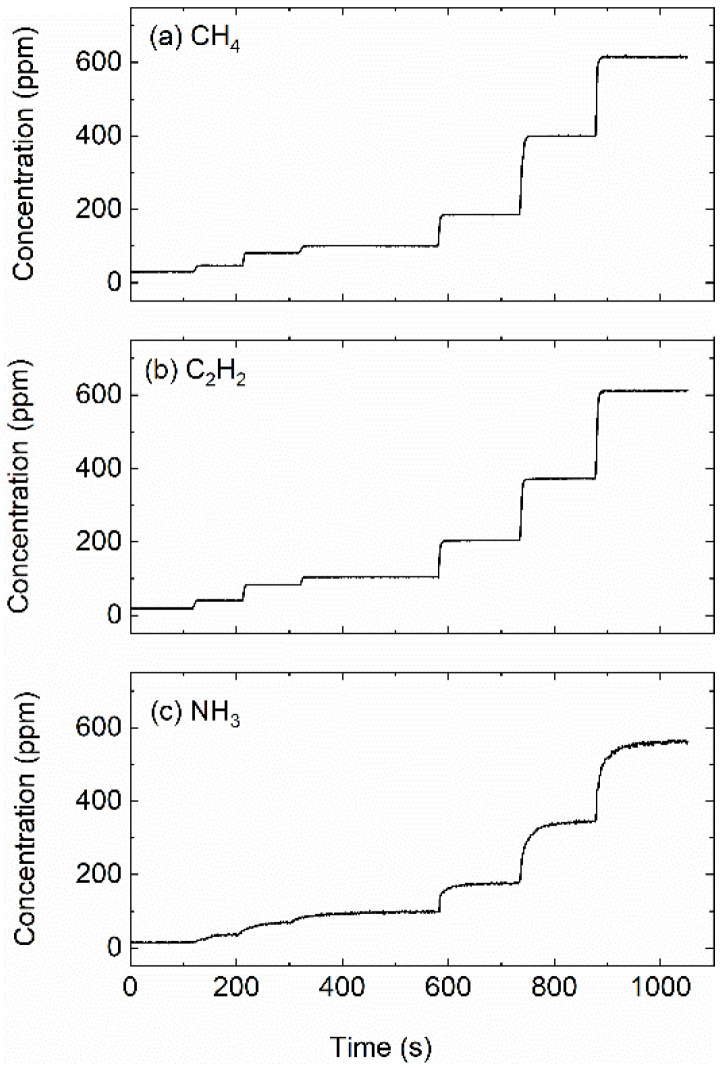
Gas flow test of the sensor for CH_4_ (**a**), C_2_H_2_ (**b**) and NH_3_ (**c**) at different concentrations.

The sensor’s dynamic performance was also examined by monitoring varying gas concentrations (20–600 ppm) under gas flowing conditions. Figure 9 presents the measured time-resolved gas concentrations. The variations of gas concentration were successfully captured. More specifically, the rapid response of the measured CH_4_ and C_2_H_2_ was observed when the gas concentration increases. However, increase tendency is evidently slower for NH_3_ within the gradient region. This is because NH_3_ is a sticky gas and some amount of gas has been stuck to the wall of the gas cell during the gas loading. Considering the linearity, precision, long-term stability and dynamic performance, the sensor can provide simultaneous measurement of CH_4_, C_2_H_2_ and NH_3_ with sub-ppm-level sensitivity and dozens of ppb-level MDL.

There exists potential interference due to the absorption overlap when measuring NH_3_ concentration with the presence of high C_2_H_2_ concentration. Take the C_2_H_2_ concentration of 200 ppm, 500 ppm and 1000 ppm, for example, detection limit of NH_3_ is estimated to be ~0.5 ppm, ~1.2 ppm and 2.6 ppm at 1 s response time. For measurement of 100 ppm NH_3_, the measurement precision will remain the constant because the overlap near the peak absorbance is negligible if the noise level is unchanged. For measuring lower concentrations (e.g., 10 ppm), the precision will deteriorate from ~0.5% to 1–2%. For the simultaneous measurements of three gases, CH_4_ measurements will not be influenced by C_2_H_2_ and NH_3_ interference because there is no absorption overlap over the entire CH_4_ features. The interference will be non-negligible only when measuring C_2_H_2_ and NH_3_ under the condition that one specie concentration is over 100 times higher than the other. However, such interference can be quantitatively determined by using the multispectral fitting method to include the multiple absorption features of C_2_H_2_ and NH_3_.

In future applications, we mainly focus on the measurement at relatively mild condition, where the trace combustion exhaust and fuel leakage are emitted to the atmospheric environment. Note that the environmental fluctuations may drift the laser wavelength and fluctuate the laser intensity. In the current work, we used scanned-wavelength modulation spectroscopy to mitigate the effect of laser wavelength drift and 2*f*/1*f* WMS strategy to eliminate the influence of received transmitted laser power. The measurement errors were observed to be within 1% for the gas concentration of 1–1000 ppm in the presence of the environmental fluctuations based on repetitive experiments on different days. Therefore, we consider the environmental fluctuations have negligible effects on measurement errors.

## 5. Conclusions

In this work, we presented a sensitive and precise laser absorption sensor for sub-ppm detection of CH_4_, C_2_H_2_ and NH_3_ by implementing NIR DFB lasers and compact multipass cell. A systematic investigation of absorption lines within NIR and MIR region was conducted and absorption lines near 6049.96, 6534.36 and 6612.71 cm^−1^ were selected for CH_4_, C_2_H_2_ and NH_3_ detection, respectively. The selected lines are adequately immune to the interferences from other species and have sufficient line-strength to ensure desired detectivity at ambient temperature and atmospheric pressure. First-harmonic-normalized wavelength modulation spectroscopy with the second harmonic detection (WMS-2*f*/1*f*) is used to ensure the robustness of measurement. Under the optimal modulation depth, good linear response (R^2^ > 0.997) was achieved within the gas concentration range of 1–1000 ppm. The multispecies sensor is estimate to have a precision of 0.5% for 30 min continuous measurement of 100 ppm gas. MDLs of 0.32 ppm, 0.16 ppm and 0.23 ppm were found at 1 s time resolution for CH_4_, C_2_H_2_ and NH_3_, respectively. By Allan–Werle deviation analysis, an improved MDL of 20–34 ppb can be found after integration time of 60–148 s. The sensitivity can be further improved by using the line-locked technique [43], adopting the hardware lock-in amplifier with higher time constant, or using advanced filtering techniques [44]. Besides the sub-ppm sensitivity and detectivity, the sensor was further demonstrated in flow gas conditions. Future work will involve the application of the sensor for monitoring the combustion exhaust from internal engines (e.g., natural gas engine, diesel engine) and further development of an FPGA-based, portable multispecies sensor.

## Data Availability

Not applicable.
